# Bladder Necrosis Associated with Placenta Accreta, Embolization, and Repair of Cystotomies

**DOI:** 10.1089/cren.2015.29007.wjw

**Published:** 2015-10-01

**Authors:** Wayland J. Wu, Arthur D. Smith, Zeph Okeke

**Affiliations:** Smith Institute for Urology, North Shore Long Island Jewish Health Systems, New Hyde Park, New York.

## Abstract

Bladder necrosis is an unusual and potentially devastating complication of embolization of the hypogastric arterial branches. The rich collateral blood supply makes this an extremely rare event. We present the case of a patient with bladder necrosis following placenta accreta that was treated with total abdominal hysterectomy and uterine artery embolization and cystotomy repairs.

## Clinical History

A thirty-year-old woman, gravida 4 para 3, with a history of placenta accreta requiring total abdominal hysterectomy and intraoperative uterine artery angioembolization to control hemorrhage during cesarean section. Her operation was complicated by several cystotomies during dissection of the uterus from the bladder. The bladder was inspected by performing a transverse incision into the dome of the bladder. Anterior and posterior cystotomies were closed in two layers using absorbable sutures and an 18-French Foley catheter was left indwelling after an effective leak testing of the bladder repair. She was transfused and then transferred to the intensive care unit. After a brief stay in the intensive care unit, she was transferred to a surgical ward and eventually discharged home with an indwelling catheter.

Ten days after discharge, a cystogram was performed without evidence of contrast extravasation, her catheter was removed. Thereafter, she developed severe dysuria ∼14 days after catheter removal and was found to have a urinary tract infection based on urine culture. Despite the use of several culture-specific antibiotics and phenazopyridine, her symptoms did not abate and the infection did not clear. She presented to the office ∼1 month after her hysterectomy for further evaluation.

## Physical Examination

General examination revealed a young, overweight, well-appearing woman in no apparent distress. Her abdomen was soft, nondistended, midline wound healing well with minimal tenderness. A genitourinary examination demonstrated a gray-colored tissue emanating from her urethral meatus. The tissue elicited tenderness with gentle traction. There was no evidence of involvement of her vagina. The remainder of her examination was unremarkable.

## Intervention

**FIG. 1.**
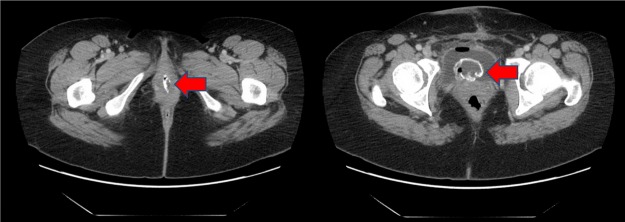
CT abdomen and pelvis axial section. High attenuation lesion in the urethra and bladder lumen (*arrow*).

**FIG. 2.**
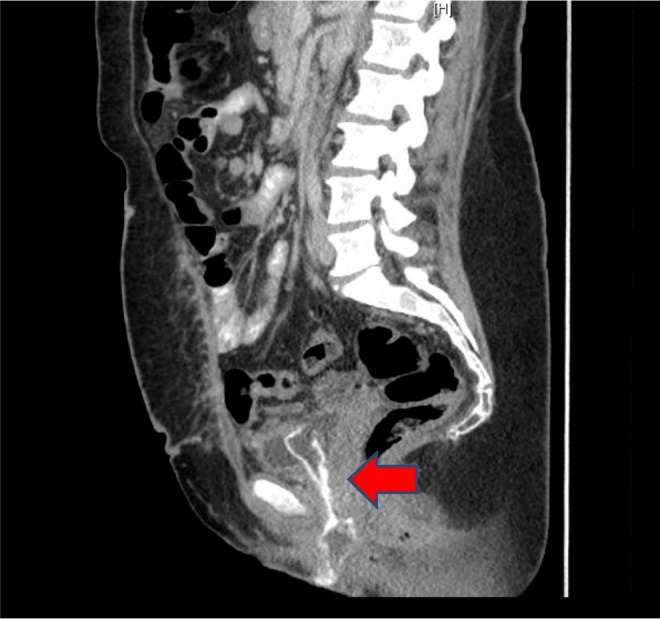
CT abdomen and pelvis sagittal section. High attenuation lesion in the urethra and bladder lumen (*arrow*).

**FIG. 3.**
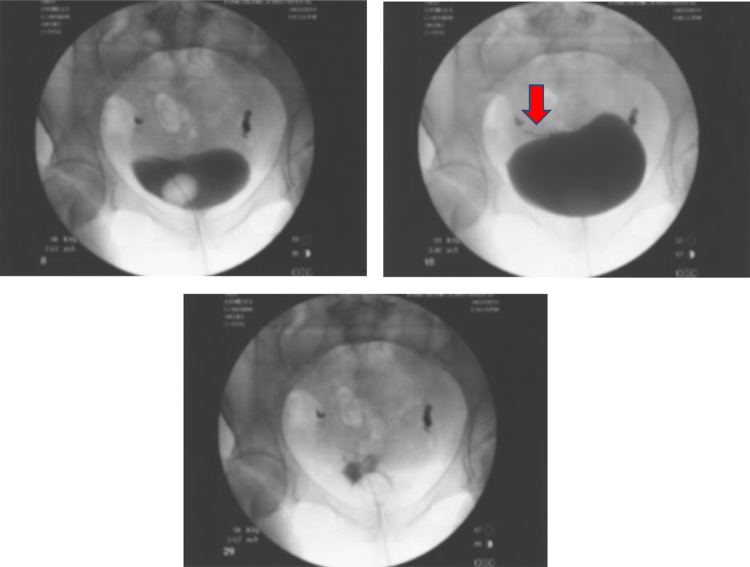
Representative images from intraoperative cystogram. A small wisp of contrast extravasation (*arrow*).

For proper evaluation of the lesion, CT of the abdomen and pelvis was performed (Figs. 1 and 2). There was a highly attenuated lesion, which was described as necrotic tissue in the bladder lumen. The patient was brought to the operating room for formal evaluation through cystoscopy under anesthesia. After induction of anesthesia, a thorough genitourinary examination was performed. A gray-colored piece of tissue was measured to be 2–3 inches in length and not easily removed by traction. There was no evidence of lesions in the vaginal vault. A 21-French rigid cystoscope was introduced into the urethral meatus and passed alongside the soft tissue mass. Upon entry into the urinary bladder, the tissue appeared to be adherent to the dome of the bladder. The lesion was resected with cold cup biopsy forceps and sent for histologic analysis. Small areas of necrosis adjacent to the lesion in question were also resected. The remainder of the bladder appeared to have normal mucosa without evidence of perforation. An 18-French Foley catheter was placed and an intraoperative cystogram was performed using fluoroscopy (Fig. 3). A small area of extravasation was noted at the dome of the bladder during the filling phase. The Foley catheter was left in place and the patient was transferred to the recovery room without complications. She was discharged the same day following her surgery.

## Outcome

**FIG. 4.**
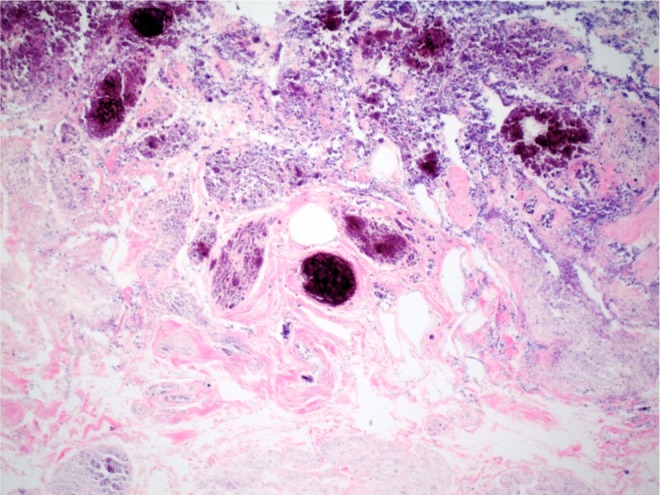
Necrotic bladder wall with ghost outlines of blood vessels and muscle bundles within lamina propria with surface dystrophic calcifications. Hematoxylin and eosin, original magnification 40×.

The patient was seen again ∼3 weeks after her surgery. A cystogram was once again performed without evidence of contrast extravasation. The urinary catheter was removed and she was able to void spontaneously. Urine culture obtained at that time was negative. She was seen again 1 week afterward for an interval follow-up. She had complete resolution of her dysuria. In addition, she denied hematuria and irritative voiding symptoms. Her abdominal examination was benign. Urine culture was again positive and appropriate antibiotics were prescribed. Final pathology demonstrated a full-thickness necrotic bladder. The piece of tissue was 12 cm in length by 0.5–2 cm in diameter (Fig. 4).

## Discussion

The bladder is richly supplied through branches of the internal iliac arteries. In this case, her history is notable for angioembolization of the uterine arteries following closure of cystotomies. There have been reports in the literature of bladder mucosal necrosis following angioembolization of the hypogastric artery branches. The repair performed, including the intentional cystotomy for inspection, may have been compromised due to angioembolization of both uterine arteries, which at times gives rise to the inferior vesical artery. A similar case was reported by Marin-Sanchez et al. Their experience described a young female patient with severe postpartum hemorrhage following vaginal delivery refractory to conservative management. Uterine artery embolization was performed and the patient presented ∼2 weeks later with a systemic inflammatory response syndrome and was found to have uterine, vaginal, and bladder necrosis. They also report this patient had necrosis at the bladder dome.^1^ She developed a vesicovaginal fistula following conservative management of her bladder necrosis, which was surgically corrected. Pisco and colleagues also reported on a male patient with bladder ischemia with desquamation of bladder mucosa following angioembolization of prostatic arteries in a series of 250 patients available for follow-up.^2^ The article did not describe the presenting symptoms that lead to suspicion of bladder wall necrosis. The patient had a bladder mass removed surgically (the article did not specify the surgical approach) and had no further complications. Ali and associates described a severe case of bladder necrosis following embolization to control hemorrhaging in a patient who suffered a pelvic fracture. Bilateral internal iliac artery angioembolization was performed complicated by bladder ischemia requiring excision and urinary diversion.^3^ These reports are similar to our case, as our patient became symptomatic in ∼3 weeks following angioembolization. Unlike the cases described, the necrotic bladder tissue prolapsed out of the urethra. Overall, bladder ischemia is a rare complication for transcatheter arterial embolization. Urologists should entertain this diagnosis for patients presenting with lower urinary tract symptoms with a recent history of angioembolization of pelvic vasculature.

## Abbreviations Used

CT: computed tomography
